# Association between Perioperative Parameters and Cognitive Impairment in Post-Cardiac Surgery Patients

**Published:** 2015-04-03

**Authors:** Saba Ghaffary, Azita Hajhossein Talasaz, Padideh Ghaeli, Abbasali Karimi, Abbas Salehiomran, Alireza Hajighasemi, Payvand Bina, Sayeh Darabi, Arash Jalali, Mehrnoush Dianatkhah, Maryam Noroozian, Nazila Shahmansouri

**Affiliations:** 1 *Department of Pharmacotherapy, Faculty of Pharmacy, Tehran University of Medical Sciences, Tehran, Iran.*; 2 *Tehran Heart Centre, Tehran University of Medical Sciences, Tehran, Iran.*; 3 *Roozbeh Hospital, Tehran University of Medical Sciences, Tehran, Iran.*

**Keywords:** Cognition disorders, Cardiac surgical procedure, Pulmonary disease, chronic obstructive, Creatinine

## Abstract

**Background:** Postoperative cognitive dysfunction (POCD) has been an important complication of cardiac surgery over the years. Neurocognitive dysfunction can affect quality of life and lead to social, functional, emotional, and financial problems in the patient’s life. To reduce POCD, we sought to identify the association between cognitive dysfunction and perioperative factors in patients undergoing cardiac surgery.

**Methods:** One hundred one patients aged between 45 and 75 years undergoing elective cardiac surgery were enrolled in this study. All the surgeries were performed on-pump by the same medical team. A brief Wechsler Memory Test (WMT) was administered before surgery, 3 to 5 days after the surgery, and 3 months after discharge. All related perioperative parameters were collected in order to study the effect of these parameters on the postoperative WMT scores and WMT score change.

**Results:** The study population consisted of 101 patients, comprising 14 (13.8%) females and 87 (86.2%) males aged between 45 and 75 years. In univariate analysis, the baseline WMT score, serum levels of lactate dehydrogenase and T3, cross-clamp time, and preexistence of chronic obstructive pulmonary disease showed significant effects on the postoperative WMT score (p value < 0.05), whereas only the baseline WMT score and chronic obstructive pulmonary disease showed strong effects on the postoperative WMT score in the multiple regression model. In addition, the multiple regression model demonstrated a significant association between the baseline WMT score, serum creatinine level, and nitrate administration and the WMT score change.

**Conclusion:** Our study showed that preexisting chronic obstructive pulmonary disease and preoperative high serum creatinine levels negatively affected cognitive function after surgery. In addition, there was a strong relationship between the patients’ basic cognition and POCD. Preoperative nitrate administration led to a significant improvement in POCD. It is also concluded that the preoperative administration of specific medicines like nitrates can reduce neurological complications after cardiac surgery.

## Introduction

Although surgical techniques and effective brain protection strategies have been improved, the incidence of neurological complications after cardiac surgery has remained relatively constant. These complications have been considered as important causes of morbidity and mortality over the years.^1^ Neurological complications after cardiac surgery are classified into Type-I and Type-II by the American College of Cardiology and the American Heart Association.^2^ Stroke, transient ischemic attack, coma, and fatal cerebral injury are categorized among Type-I neurological complications. Delirium and postoperative cognitive dysfunction (POCD), including deficits of memory, concentration, and psychomotor speed, are regarded as Type-II neurological deﬁcits.^3^ The incidence of cognitive dysfunction is reported to be highest at discharge (approximately 50 to 80%). Most of these cognitive dysfunctions are reversible between 6 weeks to 6 months after the surgery. This incidence is decreased 20 to 50% and 10 to 30% in 6 weeks and 6 months, respectively.^4^ Long-term changes such as difficulty in calculation, directions, and complex actions can also be seen in this population. Selnes et al.^5^ demonstrated that although cognitive performance improves during a year after coronary artery bypass graft surgery (CABG), it starts to decline from one to 5 years and the incidence of impairment reaches 42% after 5 years. In addition, neurocognitive dysfunction can affect quality of life and lead to social, functional, emotional, and financial problems in the patient’s life.^6^

Until recently, different mechanisms based on physiological disturbances caused by cardiopulmonary bypass (CPB) were thought to be responsible for POCD. For all the relevant studies available, however, the exact pathophysiology of neurological complications after CABG has not been clearly determined.^7^ Factors relating the existence and severity of POCD are known as perioperative parameters, which are referred to as pre-, intra-, and postoperative factors.^8^ The preoperative parameters include age, level of education, preexisting cognitive impairment, previous neurodegenerative disorders, genetic factors, and comorbidities such as diabetes mellitus, systemic hypertension, and chronic kidney disease.^9^

Different theories have been suggested for intraoperative mechanisms and pathophysiology of POCD. The formation of cerebral microemboli is one of these mechanisms. The second theory for POCD is the alterations in the cerebral perfusion and oxygenation during surgery. When the cerebral perfusion pressure is within the normal range, the cerebral blood flow is independent from the perfusion pressure. However, if autoregulation fails, the cerebral blood flow becomes pressure-dependent. The mean arterial pressure is decreased during CPB. This may be below the lower limit of autoregulation in patients with a history of hypertension.^10^ The above-mentioned cascade can give rise to change in the cerebral perfusion, thereby rendering the brain susceptible to ischemia. A reduction in the cerebral blood flow may be followed by cerebral edema^8^ and consequently impairment in the cerebral blood perfusion and oxygenation.^7^ Inflammation is one of the main mechanisms of POCD. Blood exposure to the non-endothelial surfaces during CPB activates a variety of inflammatory mediators such as coagulation cascade, fibrinolytic system, complement systems, free radicals, and factors that cause the deterioration of the preexistent lesions.^8^ Another mechanism presumed to be responsible for POCD is temperature change insofar as postoperative hyperthermia can lead to the release of free radicals and neurotransmitters in toxic amounts.^11^ Also it can increase the hematoencephalic barrier permeability and enlargement of the ischemic areas.^12^ Anesthesia, hyperglycemia, and duration of surgery are some other mechanisms suggested for POCD.^8^ Speed of CPB rewarming temperature and hypoxia after surgery are the postoperative factors associated with POCD.^13^

 Due to the clinical importance of cognitive dysfunction after cardiac surgery and its influence in the patients’ lives, we sought to find any possible association between neurocognitive function and different perioperative parameters. In addition to the postoperative Wechsler Memory Test (WMT) score, we herein report the difference between the pre- and postoperative WMT scores (WMT score change) in order to eliminate the effect of the preexisting cognition dysfunction role on the baseline WMT score and subsequently the postoperative WMT score. The aim of this study was to predict parameters that can reduce or prevent cognitive impairment and its consequences in patients undergoing cardiac surgery.

## Methods

After institutional review board approval, 101 patients undergoing elective cardiac surgery between March 2013 and March 2014 in Tehran Heart Center were enrolled in this study. Isolated CABG was performed in 82 (81%) patients, CABG plus valve replacement in 10 (10%), and valve or other types of surgery in 9 (9%). All patients younger than 45 and older than 75 years old; patients with a history of symptomatic cerebrovascular disease, stroke, seizure, psychiatric illness, and active liver disease; and those who were illiterate or innumerate were excluded from enrollment. All the surgeries were performed by the same medical team, and all of them were on-pump. Demographic information, past and present medications, history of illnesses, all required laboratory tests, administered medications in all parts of hospitalization, special techniques, and condition of surgery per patient were collected for analysis. The patients' anxiety was controlled by short-acting benzodiazepines such as Lorazepam and Oxazepam. Delirious patients were excluded from the study. In addition, patients with unexpected post-surgery complications and more than usual duration of the intensive care unit (ICU) admission (the minimum duration of ICU admission was 24 hours) were excluded from the study.

A brief neurocognitive test battery was administered before surgery and 3 to 5 days after surgery. A 3-month follow-up period was set for the patients, who were evaluated with the same test in a quiet room in the follow-up clinic of the hospital. First, the WMT was explained in detail to the participants. Assessments were performed individually by the same researcher (a trained resident of pharmacotherapy). The subtest of the WMT was revised for the patients.^6^ The WMT assesses the following in patients:

1) awareness of personal and daily tasks; 2) orientation to time and place; 3) ability of mind control with three parts of different digits count and months order in a year; 4) ability to recall the details of a short story immediately after it is read to them; 5) ability to count digits in different forward and reverse orders; 6) ability to remember the association between words after they are read to them; and 7) ability to reproduce a series of geometric shapes after a 10-second exposure from memory.^6^

The continuous variables are presented with mean and standard deviation (SD), and their associations with the postoperative WMT score and the WMT score change are reported through the Pearson correlation coefficient. The categorical variables are expressed with frequency and percentage, and their relationships with the WMT score change and the postoperative WMT score were tested through the Student t or Mann-Whitney U test. Variables with a p value < 0.2 in the univariate analysis were candidated to enter the multivariable model. A backward stepwise linear regression model was applied to find the multiple predictors of the postoperative WMT score (model A) and the WMT score change (model B). For the statistical analyses, the statistical software SPSS version 19.0 for Windows (SPSS Inc., Chicago, IL) was used.

## Results

**Figure 1:**
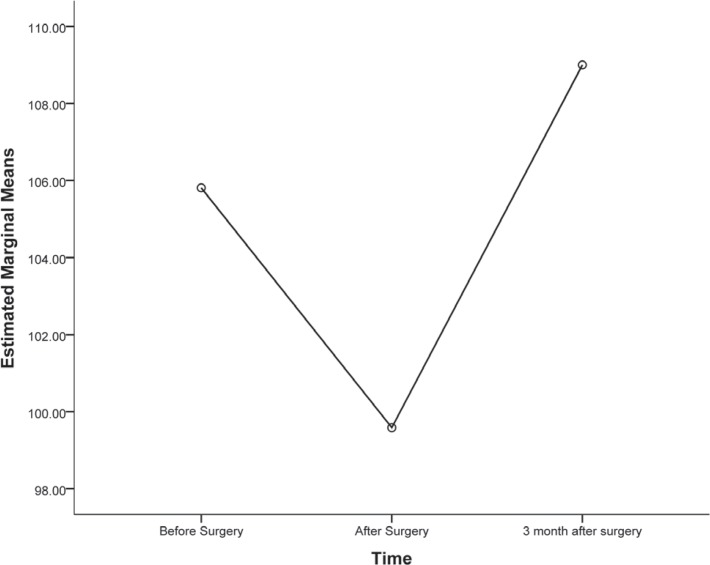
Wechsler Memory Test (WMT) scores at base line, postoperative, and 3-month follow-up evaluations

The study population consisted of 101 patients, comprising 14 (13.8%) females and 87 (86.2%) males. Among the 101 patients, 58 (57.4%) were available for the 3-month postoperative WMT follow-up. As is shown in Figure 1, although cognitive decline was evident in 65 (64.4%) patients at discharge, almost 100% of cognition function improved to baseline level after 3 months. Because of the importance of the postoperative WMT score in the prediction of long-term complications and numbers of patients on follow-up, the pre-and postoperative WMT scores and their change were analyzed in this study. All the univariate analyses are depicted in Tables 1 and 2. From the parameters included in the univariate analyses, the following showed significant effects on the postoperative WMT score (p value < 0.05): baseline WMT score; serum lactate dehydrogenase (LDH) level; serum level of T3; cross-clamp time; and preexistence of chronic obstructive pulmonary disease (Tables 1 and 2). In addition, among the preoperative medications, antiplatelet and statin administration exhibited a positive, albeit not statistically significant, effect on the postoperative WMT score (p value > 0.05).

In order to find the association between the perioperative parameters and the postoperative WMT score, first regression model (A) was conducted. In this model, the outcome was the postoperative WMT score. The characteristics of model (A) consist of an adjusted R square of 56.4%. Multiple predictors for the postoperative WMT score were baseline WMT score and preexisting chronic obstructive pulmonary disease (Table 3).

The WMT score change and perioperative parameters were compared in the univariate analyses. As is demonstrated in Tables 1 and 2, the baseline WMT score, preexisting hypertension, and preoperative nitrate administration demonstrated significant effects on the WMT score change (p value < 0.05).

Secondly, in order to find the association between the perioperative factors and the WMT score change, regression model (B) was designed. The regression model (B) characteristics consist of an adjusted R square of 21.2%. This time, the outcome of model (B) was the WMT score change. Multiple predictors for the WMT score change were the baseline WMT score, level of serum creatinine (SCr), and preoperative administration of nitrates (Table 3). 

**Figure 2:**
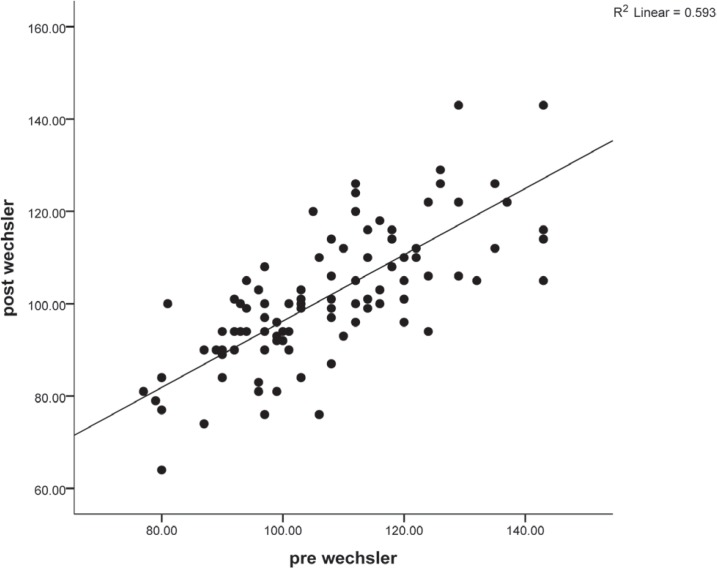
Association between the baseline Wechsler Memory Test (WMT) score and the postoperative WMT score

According to the analysis, the baseline WMT score was the strong predictive factor in both regression models. As is seen in Figure 2, there was a high correlation between the baseline and postoperative WMT scores (r = 0.77) (Figure 2).

**Table 1:** Association between the qualitative variables and the postoperative WMT score and WMT score change (n = 101)

	Number	Postoperative WMT Score		WMT Score Change	
		Mean±SD	P value	Mean±SD	P value
Demographic variables					
Sex					0.150
Male	87	102.7±16.3	0.511	-6.8±11.2	
Female	14	99.6±13.6		-1.6±10.5	
Alcohol consumption					0.185
Yes	17	106.8±17.1	0.271	-10.4±12.8	
No	84	101.6±15.7		-5.3±10.8	
Opium addiction					0.709
Yes	15	101.8±18.2	0.931	-6.1±11.2	
No	86	101.4±15.1		-7.6±11.9	
Risk factors					
Current smoker					0.317
Yes	14	106.5±16.6	0.264	-5.6±11.3	
No	87	101.4±15.7		-9.1±11.1	
Positive family history					0.769
Yes	46	100.4±15.3	0.449	-5.8±11.2	
No	55	102.9±16.4		-7.0±11.4	
Dyslipidemia					0.147
Yes	48	104.3±17.1	0.216	-4.6±11.9	
No	53	100.2±14.8		-7.7±10.5	
Hypertension					0.046
Yes	49	102.3±15.1	0.895	-3.9±10.2	
No	52	101.8±16.9		-8.8±11.7	
Diabetes					0.899
Yes	34	102.7±12.9	0.861	-6.3±10.5	
No	67	102.1±17.4		-5.8±12.7	
Comorbidities					
Renal failure					0.094
Yes	2	90.0±8.5	0.279	-5.9±11.3	
No	99	102.5±16.1		-17.5±2.1	
COPD					0.623
Yes	16	94.5±9.6	0.047	-5.8±11.3	
No	85	102.9±15.9		-8.7±13.1	
Myocardial infarction					0.601
Yes	27	101.3±16.3	0.798	-5.9±11.1	
No	74	102.3±15.9		-7.3±11.6	
Chronic heart failure					0.810
Yes	5	98.4±19.2	0.584	-6.3±11.3	
No	96	102.4±15.9		-3.4±10.9	
Preoperative medications					
Beta blocker					0.886
Yes	83	101.2±16.4	0.276	-6.4±10.1	
No	18	106.3±12.4		-6.3±11.4	
Calcium channel blocker					0.594
Yes	14	101.2±10.1	0.871	-6.0±10.9	
No	87	102.2±16.7		-8.1±12.6	
Nitrates					0.019
Yes	82	102.7±16.2	0.266	-0.5±8.9	
No	19	97.7±14.3		-7.4±11.3	
Diuretics					0.275
Yes	22	101.7±15.6	0.744	-5.4±10.5	
No	79	102.9±17.5		-9.3±13.1	
ACEI or ARB					0.835
Yes	70	101.1±16.7	0.327	-5.7±10.2	
No	31	104.6±13.7		-6.6±11.6	
Antiplatelets					0.750
Yes	5	113.0±14.2	0.113	-6.4±11.4	
No	96	101.4±15.9		-4.4±6.7	
Anticoagulants					0.458
Yes	26	100.2±18.1	0.519	-6.9±11.6	
No	75	102.6±15.2		-4.8±10.2	
Aspirin					0.145
Yes	78	101.3±14.2	0.448	-2.8±11.6	
No	23	104.5±21.9		-7.2±11.1	
Steroids					0.493
Yes	2	113.0±18.4	0.326	-6.4±11.2	
No	99	101.7±15.9		-1.0±11.3	
Digoxin					0.870
Yes	5	98.2±16.3	0.590	-6.3±11.4	
No	96	102.2±15.9		-6.4±8.6	
Amiodarone					0.780
Yes	2	94.5±3.5	0.506	-6.4±11.3	
No	99	102.1±16.1		-3.5±4.9	
Oral anti-hyperglycemic agents					0.528
Yes	17	102.7±14.6	0.912	-5.8±10.8	
No	84	102.2±16.3		-7.6±13.5	
Insulin					0.622
Yes	15	100.4±10.9	0.618	-6.02±11.1	
No	86	102.6±16.8		-6.9±12.9	
Statins					0.667
Yes	89	102.9±16.4	0.193	-4.5±9.8	
No	12	95.5±9.5		-6.3±11.5	
Bronchodilator agent					0.313
Yes	12	104.0±15.1	0.683	-5.4±10.8	
No	89	101.9±16.2		-11.2±14.3	
Intraoperative parameters					
Isolated CABG					0.746
Yes	82	103.1±15.1	0.096	-8.0±12.7	
No	19	95.7±19.2		-6.0±10.9	
Left main disease					0.928
Yes	4	89.5±9.8	0.111	-6.3±11.4	
No	97	102.5±15.9		-6.0±7.4	
Preoperative AF					0.799
Yes	4	91.2±10.7	0.171	-6.3±11.3	
No	97	102.4±16.1		-7.2±9.7	

WMT, Wechsler Memory Test; COPD, Chronic obstructive pulmonary disease; ACEI, Angiotensin-converting enzyme inhibitor; ARB, Angiotensin receptor blocker; CABG,  Coronary artery bypass graft; AF, Atrial fibrillation

**Table 2:** Association between the quantitative variables and the postoperative WMT score and WMT score change.

Quantitative Perioperative Parameters	Postoperative WMT Score		WMT Score Change	
Variable	r	P value	r	P value
Pre-WMT score	0.770	< 0.001	0.488	< 0.001
Demographic variables Age BMI (kg/m^2^)	-0.126 -0.491	0.213 0.628	-0.781 0.024	0.443 0.812
Baseline lab data Platelet count (count/µL) Neutrophil count (cells/µL) Lymphocyte count (cells/µL) LDH (U/L) CPK (U/L) Magnesium (mEq/L) TSH (µIU/mL) T3 (ng/ml) T4 (ng/ml) White blood cells (cells/µL) hemoglobin (g/dL) FBS (mg/dL) Serum creatinine (mg/dL) Total cholesterol (mg/dL) HDL (mg/dL) LDL (mg/dL) Triglyceride (mg/dL)	0.068 -0.451 0.088 -0.284 0.026 -0.153 0.047 0.449 0.086 0.003 -0.002 0.113 -0.091 -0.167 -0.005 -0.177 0.011	0.499 0.659 0.389 0.005 0.800 0.131 0.795 0.009 0.635 0.977 0.981 0.265 0.365 0.097 0.964 0.078 0.910	0.072 -0.086 0.150 0.039 -0.139 0.181 -0.162 0.196 0.188 0.002 0.069 -0.149 -0.172 0.065 -0.052 0.014 0.155	0.478 0.398 0.140 0.707 0.173 0.073 0.367 0.273 0.295 0.986 0.496 0.141 0.087 0.523 0.609 0.892 0.124
Intraoperative parameters Pump time (Minute) Cross-clamp time (Minute) Total graft	-0.181 -0.204 0.007	0.077 0.045 0.948	0.071 0.009 0.009	0.497 0.930 0.930

WMT, Wechsler memory test; BMI, Body mass index; LDH, Lactate dehydrogenase; CPK, Creatine phosphokinase; TSH, Thyroid-stimulating hormone; FBS, Fasting blood sugar; HDL, High-density lipoprotein; LDL, Low-density lipoprotein

**Table 3:** Multiple predictors of the postoperative WMT score and WMT score change*

	Regression. Coefficient (95% CI)	P Value
postoperative WMT score		
Intercept	32.988 (8.10 to 57.88)	0.012
Preoperative WMT score	0.633 (0.41 to 0.86)	< 0.001
Chronic obstructive pulmonary disease	-16.323 (-30.25 to -2.40)	0.024
WMT score change		
Intercept	28.208 (14.31 to 42.11)	< 0.001
Preoperative WMT score	-0.236 (-0.36 to -0.11)	< 0.001
Preoperative serum creatinine	-4.121 (-7.99 to -0.26)	0.037
Preoperative nitrates	-6.299 (-12.40 to -0.19)	0.043

WMT, Wechsler memory test

* Adjusted R square is 56.4% and 21.2% for models (A) and (B), respectively

## Discussion

In this study, we identified positive and negative associations between perioperative parameters and memory impairment following cardiac surgery in order to prevent POCD and its long-term complications. Our results showed that the patients followed the same pattern of cognitive impairment (postoperatively and 3 months after surgery) as that reported in previous studies.

Our results revealed a positive, albeit not significant, relationship between anti-lipid agents (statins) as preoperative medications and POCD. Previous studies have mentioned the positive effects of preoperative statin therapy on the incidence of atrial fibrillation, stroke, delirium, and mortality after cardiac surgery.^14^ Inflammation is one of the primary etiologies of POCD.^15^ Statins reduce the different processes of inflammation by reducing proinﬂammatory cytokines release and neutrophil adhesion and upregulating the secretion of anti-inﬂammatory cytokines after cardiovascular surgery, which is one of the primary etiologies of POCD. Recent studies have noted that statins increase the cerebral blood flow, restrict the permeability of the blood-brain barrier, and decrease the transmigration of leukocytes.^15^ Due to the mentioned mechanisms, statins are effective agents in reducing cardiovascular surgical complications and have neuroprotective effects.^16^ Previous studies have shown that POCD can be affected by the degree of aortic atherosclerosis and non-coronary manifestation of atherosclerosis.^17^ The atheroma of the aortic wall can be the origin of some embolisms.^8^ Heyer et al*.*^18^ reported a neurocognitive decline in patients undergoing carotid artery surgery. As well as the mechanisms mentioned in previous studies, our statistical analysis demonstrated a reverse relationship between the *low-density lipoprotein* (LDL) level and postoperative cognitive impairment.

Among the intraoperative parameters, pump time and cross-clamp time showed a reverse effect on the postoperative WMT score. This is in line with physiological dysfunction, followed by the CPB technique.^7^

*Lactate dehydrogenase* (LDH) plays an important role in cellular respiration. LDH is released into the bloodstream when tissues are damaged. It is also increased after myocardial and neuronal damage.^19^ The results showed that the patients with higher serum levels of LDH had significantly more memory impairment after surgery. However in regression model (A), no strong relation was found between the serum level of LDH and the postoperative WMT score.

Another parameter that showed a significant association with the postoperative WMT score was the serum level of T3. Although the T3 level decreases after cardiac surgery with CPB, the concentrations of T4 and thyroid-stimulating hormone (TSH) stay normal. The patients with low cardiac output syndrome along with those who had lower serum T3 levels prior to CPB showed significantly lower T3 concentrations after surgery. CPB leads to low serum T3 level syndrome up to 3 days after surgery.^20^ Thyroxine increases cognitive performance following cholinergic function enhancement.^21^ Triiodothyronine can confer vasodilation and relaxation via a direct effect on vascular smooth-muscle cells. Thyroid hormones increase renin release and activate angiotensin–aldosterone axis and, consequently, raise the plasma volume. Subsequently, cardiac output increases after CABG.^22^ The mentioned hypothesis may be justifiable with the enhancement of the blood circulation and improvement in oxygenation in the presence of higher values of the serum T3 level.

Our analysis in regression model (A) showed that preexisting chronic obstructive pulmonary disease could be a predictor of the postoperative WMT score reduction. In parallel to our study, Dodd et al.^23^ reported that 77% of their patients with chronic obstructive pulmonary disease had cognitive impairment. This category of patients is at increased risk of neuronal injury due to hypoxemia, coexistent vascular disease, and smoking and is, thus, at a higher risk of postoperative cognitive impairment.

Although cognitive impairment and dementia are common in chronic kidney disease patients, the exact mechanisms remain unclear. These patients are prone to increased prevalence of vascular dementia. Some studies have suggested that multiple factors may affect cognitive impairment in patients with chronic kidney disease, including anemia, increased levels of inflammatory cytokines, oxidative stress, and alterations in lipid and homocysteine metabolism. The Kurella et al.^24^ study demonstrated that an elevation in the SCr level was allied to 37% enhancement in the risk of dementia incidence. Our results showed a reverse relationship between the WMT score change and the patients’ preoperative SCr level. In spite of its p value, the final regression model (B) showed a strong association between them. The effect of the patient's SCr level on the postoperative WMT change can be explained by the different pathophysiology of chronic kidney diseases such as inflammation.

In the present study, the administration of nitrates as a preoperative medication led to a significant decrease in the WMT score change. Hence, regression model (B) showed the effective influence of nitrate administration on the WMT score change. There are some hypotheses regarding nitrate administration and cognitive function. Previous animal studies have indicated that nitrate components are effective in memory improvement owing to the following proposed mechanisms. Nitrite oxide induces the activation of soluble guanylyl cyclase (sGC) to release cyclic guanosine-2':3'-monophosphate (cGMP). In the hippocampus, nitrates modulate signaling cascades via mitogen-activated protein kinase-extracellular signal-regulated kinase and cyclic adenosine monophosphate (cAMP)-responsive element-binding protein. Consequently, modulating synaptic transmission in the brain regions is important for learning and memory.^25^ Nitrates also have some vasodilatory actions. Disturbance in the regional cerebral blood flow in patients with a hypertension history leads to performance impairment in verbal memory tasks.^26^ Reynolds et al.^27^ proved that a post-ischemia subcutaneous administration of nitrate components dramatically decreases the brain infarct volume. According to the mentioned mechanisms and studies, nitrates are able to reduce POCD by improving thecerebral blood flow.

Our regression models demonstrated that the baseline WMT score was a significant predictor for both postoperative WMT score and WMT score change, model (A) and model (B) respectively. Given the adjusted R square of 56.4% in model (A) in comparison with 21.2% in model (B), the baseline WMT score was a stronger predictor of the postoperative WMT score than the WMT score change. In concordance with these results, previous studies have supported the idea that basic cognitive function is a predictable factor for postoperative cognition disorders owing to the high prevalence of small-vessel ischemic disease, lacunar infarctions, and other brain disorders.^9^ Previous studies have borne out the idea that patients with preexisting silent infarctions and small-vessel disease are at a higher risk of postoperative adverse neurological outcomes.^28^

Our study encountered some limitations. Although all the patients were called for follow-up, many of them failed to show up at the appointed time. In addition, the WMT score of the follow-up was reported in order to show the pattern of cognitive impairment in long term; therefore, the data of the follow-up section were not analyzed.

## Conclusion

Given the commonality of the risk factors for ischemia between brain and heart, cognition impairment following cardiac surgery can be explained by the initiation of vascular dementia, hidden dementia, or a combination of Alzheimer and atherosclerosis of the brain vessels. The diagnosis of positive and negative factors that are effective in POCD after cardiovascular surgery can decrease the patient’s cognition complications after surgery. 

In conclusion, it can be hypothesized that preexisting diseases such as chronic obstructive pulmonary disease and preoperative SCr level could render a patient prone to a higher risk of POCD. Improvement in the CPB technique by reducing the cross-clamp time and pump time may improve the patient’s cognition function after cardiac surgery. Among all the perioperative parameters, our patients’ basic cognition function played the most important role in the prediction of POCD. Among the preoperative medications, nitrate administration conferred a significant improvement in the patients' POCD and could, as such, be used as a protective agent in POCD.

## Notes:

***This paper should be cited as:*** Ghaffary S, Talasaz AH, Ghaeli P, Karimi A, Salehiomran A, Hajighasemi A, Bina P, Darabi S, Jalali A, Dianatkhah M, Noroozian M, Shahmansouri N. Association between Perioperative Parameters and Cognitive Impairment in Post-Cardiac Surgery Patients. J Teh Univ Heart Ctr 2015;10(2):85-92.
